# Energy Expenditure and Physical Activity in Recovering Malnourished Infants

**DOI:** 10.1155/2010/171490

**Published:** 2009-12-27

**Authors:** Russell Rising, Gul Tiryaki Sonmez

**Affiliations:** ^1^D & S Consulting Inc., 1 Horizon Rd, #1407, Fort Lee, NJ 07024, USA; ^2^Department of Health Sciences, Lehman College, CUNY, 250 Bedford Park Blvd West, Bronx, NY 10468, USA

## Abstract

*Background*. Malnourished infants are small for age and weight. *Objectives*. Determine profiles in 24-hour energy metabolism in recovering malnourished infants and compare to similarly aged healthy controls. *Methods*. 10 malnourished infants (58.1 ± 5.9 cm, 7.7 ± 5.6 months) were healthy prior to spending 22 hours in the Enhanced Metabolic Testing Activity Chamber for measurement of EE (kcal/min), sleeping metabolic rate (SMR; kcal/min), respiratory quotient (RQ; VCO_2_/VO_2_), and physical activity (PA; oscillations in wt/min/kg body weight). Metabolic data were extrapolated to 24 hours (kcal/kg/d). Energy intake (kcal/kg/d) and the proportions (%) of carbohydrate, protein, and fat were calculated. Anthropometrics for malnourished infants were obtained. Statistical differences (*P* < .05) between groups were determined (SPSS, version 13). *Results*. In comparison to controls, malnourished infants were lighter (4.1 ± 1.2 versus 7.3 ± 0.8 kg; *P* < .05), had less body fat % (10.3 ± 7.6 versus 25.7 ± 2.5), and lower BMI (12.0 ± 1.7 versus 15.5 ± 1.5; *P* < .05). In contrast, they had greater energy intake (142.7 ± 14.6 versus 85.1 ± 25.8; *P* < .05) with a greater percentage of carbohydrates (55.1 ± 3.9 versus 47.2 ± 5.2; *P* < .05). However, malnourished infants had greater 24-hour EE (101.3 ± 20.1 versus 78.6 ± 8.4; *P* < .05), SMR (92.6 ± 17.1 versus 65.0 ± 3.9; *P* < .05), and RQ (1.00 ± 0.13 versus 0.86 ± 0.08; *P* < .05) along with a lower amount of PA (2.3 ± 0.94 versus 4.0 ± 1.5; *P* < .05). *Conclusions*. Malnourished infants require more energy, possibly for growth.

## 1. Introduction

Malnutrition is a consequence of inadequate diet and frequent infections, leading to deficiencies in calories, protein, vitamins, and minerals. Malnutrition remains a pervasive problem in developing countries, where poverty is a strong underlying determinant. All ages are at risk, but malnutrition is most prevalent among children under five years of age, especially in the weaning and postweaning period of six to twenty-four months. The World Health Organization (WHO) has estimated that approximately 27% (168 million) of children under the age of five years are underweight [1]. Underweight children are at increased risk of mortality from infectious illnesses such as diarrhea and pneumonia [2].

In malnourished children, stunted growth and a lower amount of spontaneous physical activity are adaptations to reduced energy intake in an attempt to preserve vital functions [3]. Furthermore, other effects of malnutrition include poor brain development [4] possibly leading to poor cognition [5]. Another physiological adaptation includes a reduced acute-phase protein response to infection [6]. Finally, children with untreated malnutrition suffer from repeated episodes of acute diarrhea resulting in dehydration [7].

Only in our previous study [8] has there ever been a long-duration study (22 hours) where energy expenditure was measured continuously in healthy infants utilizing indirect calorimetry. Due to the length of the metabolic measurements, we determined that healthy infants display a metabolic circadian rhythm by four months of age [8]. At the time of this study we were the only research group in the world conducting these types of long-term energy expenditure measurements utilizing this technology. Now, we have taken our perfected methodology and applied it to infants suffering from malnutrition. This might reveal differences in the energy expenditure profile not detectable with short-duration indirect calorimetric measurements. These longer duration metabolic measurements might lead to new and improved nutrition rehabilitation regimes fine-tuned for malnourished or infants suffering from various metabolic disorders.

## 2. Materials and Methods

### 2.1. Subjects

Ten chronically malnourished full term infants (7.7 ± 5.0 months, 4.1 ± 1.2 kg, 58.1 ± 5.9 cm, 7 males and 3 females), with a birth weight of 2.7 ± 0.6 kg were recruited from the inpatient metabolic ward of the Department of Pediatrics, Federal University of Bahia School of Medicine, Hospital Universitario Professor Edgar Stantos, Salvador, Brazil. Six were classified as primary and four as suffering from secondary malnutrition as determined by a pediatrician familiar with our study. The data from these infants were compared to 10 healthy formula-fed (Carnation Good Start with iron) counterparts (7.3 ± 0.8 kg, 68.8 ± 2.8 cm, 5.0 ± 0.7 months) from a prior similar metabolic study in Miami, Florida, USA [8]. Only infants that were admitted for severe malnutrition with a diagnosis of Marasmus or Kwashiorkor were studied. No healthy infants were recruited for this study since we already had data from such a group from a prior metabolic study [8]. In our group of 10 infants, six were being treated for Marasmus and four for Kwashiorkor. Marasmus was the main reason for primary malnutrition in those infants so classified. The four infants classified as having secondary malnutrition were also being treated for Kwashiorkor. The mean admission weight for the 10 infants was 3.7 ± 1.20 kg. Moreover, there was no presence of concurrent secondary infection in those infants that were classified as having secondary malnutrition when they underwent metabolic testing. Furthermore, all infants who participated in the study were less than the 5th percentile for weight-for-length, length-for-age and weight-for-age according to the NCHS growth charts [9]. Moreover, all infants were classified as being malnourished according to weight-for-age and length-for-age z-scores being less than or equal to −2 upon admission [10]. Once identified, they were studied after they showed appropriate recovery from infectious illness as determined by the attending pediatrician. Moreover, there was no indication or presence of edema at the time of the metabolic test as verified by the pediatrician familiar with the study. This was usually three days after admission. This was also verified by the 0.36 ± 0.22 kg of weight gained between admission and the time of the metabolic test. Moreover, infants had to have a normal body temperature, as determined by the measurement of the rectal temperature, just prior to metabolic testing. Finally, infants had to be consuming the prescribed amount of formula with no presence of acute diarrhea or vomiting for at least 24 hours prior to metabolic testing. Infants were excluded if they were exclusively breastfed or presented with renal, hepatic, respiratory, and/or cardiac chronic diseases. Moreover, those with serious infections (pneumonia, meningitis, septicemia, etc.) requiring intensive care were also excluded. All infants studied were of African descent and from improvised families [11] where there were 4.5 ± 0.5 persons living within the household. The average monthly household income was 230.00 ± 133.00 US dollars.

Biological parents were provided with a complete explanation regarding the purpose, procedure, risks, and benefits of the study and informed consent was obtained from at least one parent of each infant. The study was approved by the Institutional Review Board of the Hospital Universitario Professor Edgar Stantos, Federal University of Bahia School of Medicine, and by the Institute Review Board of Lehman College, Bronx, NY.

### 2.2. Anthropometry

Length was measured in duplicate with a horizontal stadiometer (Perspective Enterprises, Kalamazoo, MI) and body weight was the average of two measurements obtained with an infant scale (Cardinal Detecto, Webb City, MO). Body composition was determined through the mean of triplicate measurements of triceps skin-fold thickness on the right side of the body using a Lange skin-fold caliper (Lange, Beta Technology, Cambridge MD) according to a standard procedure [12]. Calculation of the body fat was based on Siri's equation [body fat  % of weight  = (4.95/body density  − 4.5)  × 100], where body density is calculated from age and sex adjusted regression equations designed for malnourished infants according to Laditan and Ayeni [13]. Infant BMI (kg/m^2^) was calculated and all anthropometric measurements were made by a trained registered dietitian. Length-for-age, weight-for-age, and weight-for-length z-scores were calculated with Epi Info software (Version 3.5.1) utilizing the 1978 WHO/CDC growth reference [10].

### 2.3. Energy Intake

Total energy intake, and the percentage of carbohydrate, fat, and protein consumed, were determined by utilizing the formula manufacturer's proximate analysis (Nestle Good Start with DHA & ARA) and by the amount consumed by the infant though out the 22-hour metabolic test using calibrated infant feeding bottles [8]. The recording of energy intake started one-hour prior to and ended one-hour before the conclusion of the 22-hour metabolic testing period. The one-hour off-set was necessary to include or exclude energy consumed before and after the metabolic testing period due to the gut transient time for formula in this age infant [14]. Furthermore, none of these infants consumed energy from other sources such as solid foods or other liquid supplements during the study. All infants were formula fed for at least three months prior to metabolic measurements.

### 2.4. Twenty-Two Hour Energy Expenditure

Prior to each metabolic measurement, the Enhanced Metabolic Testing Activity Chamber (EMTAC) instrumentation was calibrated with standard gases with a known concentration of oxygen and carbon dioxide. Furthermore, parents were given instruction on how to interact with their infants and were given time to practice using the hand access ports prior to metabolic testing [8, 15–17]. Once all of the instruction and calibrations were complete each infant was placed in the EMTAC for 22 hours from 11:30 AM till 9:30 AM the following day for continuous measurements of energy expenditure (EE; kcal/min), physical activity (PA; oscillations in weight/min/kg body weight), and the respiratory quotient (RQ; VCO_2_/VO_2_). Two hours were allowed for instrument calibrations, parental instruction and practice using the hand-access ports. Any supplies such as diapers, formula, or toys were placed in the EMTAC in hanging bags before the start of the test. Parents continued to formula feed their infants during metabolic testing. Breast fed infants were not studied due to the difficulty of having pumped breast milk available for the infant during the long duration metabolic testing period. Moreover, we did not have the ability to keep breast milk cool within the EMTAC enclosure for the 22-hour metabolic test. The family of the infant was provided lodging within the laboratory during the entire testing period [8]. The investigator, pediatric research fellows, and attending pediatricians that were familiar with the study acted as observers on rotating eight-hour shifts and recorded all infant activities such as infant feedings, crying, periods of observed sleep, and amount of parental interaction during the entire 22-hour testing period.

Energy expenditure, sleeping metabolic rate, physical activity, and the respiratory quotient were continuously calculated as described in a previous infant 24-hour metabolic study [8]. The EMTAC, methodology for measurement and calculation of each component of 24-hour extrapolated EE, and correction for parental interaction have been described in previous studies [8, 15–17].

### 2.5. Calculations

To correct the continuous metabolic data for differences in body weight, each five-minute data summary period (kcal/min) was divided by kg body weight and expressed as kcal/min/kg. Next, the corrected continuous metabolic data for each infant were than divided into three separate periods consisting of 12, eight, and two hours each, respectively. The daytime period ranged from 11:30 AM till 11:29 PM, the night time period ranged from 11:30 PM till 7:29 AM the next day and the morning period ranged from 7:30 AM till the completion of the metabolic test at 9:30 AM. The criteria used for the division of the metabolic data into the specific time periods are similar to that used in previous metabolic studies in infants [8, 15–17] and in adults [18, 19].

### 2.6. Statistical Analysis

The sample size in this study was determined in advance, based on data obtained from short and long-term metabolic measurements in the EMTAC in previous studies [8, 15–17]. Moreover, the number of infants needed to detect a 5% difference in metabolic and anthropometric parameters, were then calculated according to the formula of Kuzma [20]. All data were analyzed utilizing SPSS (Version 13, Chicago, IL) and expressed as Mean ± Standard Deviation. Significance (*P* < .05) was determined at the five percent level of probability. Finally, the Shapiro-Wilk test of Normality was utilized to determine whether the distribution was normal for both the malnourished and healthy infants. The test suggests that both the malnourished (*P* < .68) and healthy infants (*P* < .15) were found to be normally distributed. Therefore, parametric statistical analysis was utilized for our data analysis.

Comparisons for 24-hour extrapolated metabolic rate and anthropometric data between malnourished and healthy infants were determined by Independent *t*-test (*P* < .05). Furthermore, within each group (malnourished and healthy) one-way ANOVA with Least Significant Difference was utilized to determine differences between each of the three time periods (day time, night time, and morning) for continuous energy expenditure (kcal/kg/min), respiratory quotients (VCO_2_/VO_2_), and physical activity (Oscillations in weight/min/kg body weight). All metabolic results are expressed as per kg/body weight unless otherwise noted.

## 3. Results

### 3.1. Anthropometry

In comparison to similarly aged healthy controls, the malnourished infants in this study were shorter, lighter, and had less body fat and fat-free mass, along with a lower BMI. Moreover, the malnourished infants had lower z-scores for length-for-age (*P* < .05) and weight-for-age (*P* < .05), in comparison to their healthy counterparts while no differences were found between the two groups for weight-for-length (Table 1). However, no differences were found between the two groups in terms of maternal BMI (Table 1) which was within the normal weight range of 20–25 kg/m^2^. Finally, the malnourished infants were living below the poverty line as shown by the monthly income of $230.00 ± $133.00 and number of persons residing in the household (4.5 ± 0.5).

### 3.2. Twenty-Four-Hour Energy Metabolism

According to Table 2, the malnourished infants consumed more energy (*P* < .05) during the metabolic test than their healthy counterparts, with a greater proportion of energy as carbohydrate (*P* < .05). Twenty-four-hour extrapolated energy expenditure (24-hour EE) and sleeping metabolic rates (SMR), along with the respiratory quotient (RQ), were greater (*P* < .05) in the malnourished infants in comparison to their healthy counterparts (Table 2). However, the malnourished infants were less physically active (*P* < .05, Table 2). Moreover, the malnourished infants cried (*P* < .05) and slept (*P* < .05) longer during metabolic testing (*P* < .05) than their healthy counterparts. Finally, there were no differences between the two groups in the amount of time parents spent interacting with their infants (Table 2). 

### 3.3. Continuous 22-Hour Energy Expenditure

**Figure 1:**
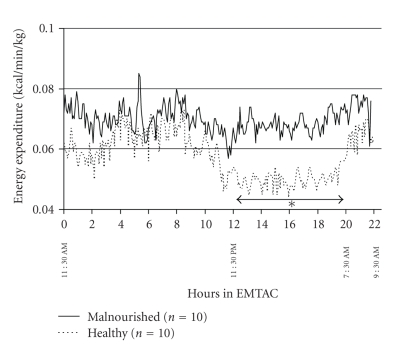
Continuous energy expenditure (kcal/min/kg) for 22 hours in 10 malnourished (solid line) compared to 10 healthy infant controls (dotted line). Each data point represents mean energy expenditure over a 5-minute period.

**Figure 2:**
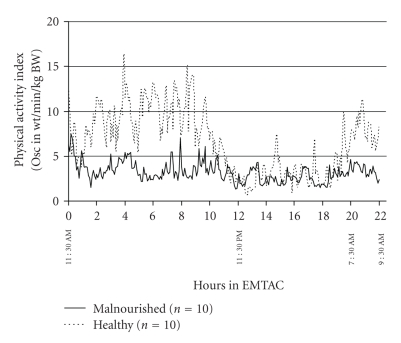
Continuous physical activity (oscillations in weight/min/kg body weight) for 22 hours in 10 malnourished (solid line) compared to 10 healthy infant controls (dotted line). Each data point represents mean physical activity over a 5-minute period.

**Figure 3:**
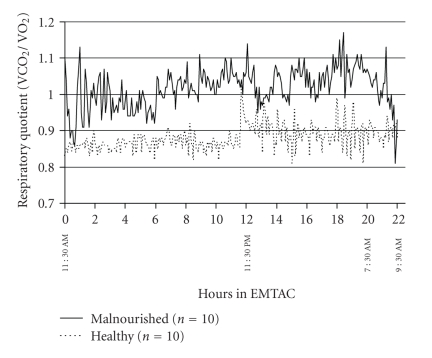
Continuous respiratory quotient (VCO_2_/VO_2_) for 22 hours in malnourished (solid line) compared to 10 healthy infant controls (dotted line). Each data point represents mean respiratory quotient over a 5-minute period.

The metabolic pattern for both groups of infants throughout the 22-hour measurement period is shown in Figure 1. The malnourished infants appeared to have greater energy expenditure during the day and morning periods in comparison to their healthy counterparts. However, during the night time period (11:30 PM till 7:30 AM) the healthy infants showed a significant (*P* < .05) decrease in energy expenditure as opposed to the lack of a similar decrease in the malnourished infants. Similar results were obtained for physical activity where the malnourished infants showed no significant changes in this parameter throughout the 22-hour metabolic test (Figure 2). However, even though not significant, the healthy infants showed greater amounts of physical activity during the day and morning periods in comparison to the night time period (Figure 2). The respiratory quotient ranged from 0.83 to 1.13 across all the malnourished infants over the course of the metabolic test. This is in comparison to the range of 0.70 to 0.99 in the healthy infants undergoing a similar study (Figure 3). During metabolic testing the respiratory quotient remained higher in the malnourished infants in comparison to their healthy counterparts.

## 4. Discussion

This is the first study of its kind where a direct comparison was made between similarly aged chronically malnourished and healthy full term infants in regards to their continuous 22-hour metabolic profiles utilizing an established accurate technology [8, 16, 17]. Moreover, at the time of the metabolic test, none of the infants had edema or were febrile which might have affected their 22-hour energy expenditure profile.

In regards to anthropometrics, we found that malnourished infants had lower body weight and length, less body fat and fat-free mass, along with a lower BMI. However, the biological parents of both groups of infants had BMI's within the normal weight range (19.1 to 27.3 kg/m^2^). Moreover, the malnourished infants were from improvised families [11, 12].

In regards to energy metabolism, malnourished infants consumed and expended more energy, had higher respiratory quotients, greater sleeping metabolic rates, and were less physically active. Moreover, they slept and cried more. Finally, they consumed a greater portion of their energy as carbohydrate.

The higher proportion of energy intake as carbohydrate in the malnourished infants might explain the higher respiratory quotients found during the 22-hour metabolic test. Respiratory quotients greater than one might reflect increased lipid synthesis. This is similar to the findings in one study where low birth-weight infants on total parenteral nutrition had greater lipid synthesis on a high carbohydrate intake of 12 g/kg/day [21]. It is possible that increased carbohydrate intake in the malnourished infants could lead to altered energy metabolism later in life. This might be due to altered insulin sensitivity created during the time of malnutrition. In one study children who were born prematurely had decreased insulin sensitivity as compared to children born at term. Moreover, an excess childhood weight gain was associated with greater insulin resistance after birth [22]. It is possible that the additional carbohydrate intake in our malnourished infants might lead to the possibility of insulin resistance and a greater possibility of excess body weight gain after their recovery from malnutrition during later childhood.

During the 22-hour metabolic test, the malnourished infants did not display any kind of a metabolic circadian rhythm. In a previous study, infants of Hispanic decent as young as 4 months displayed a metabolic Circadian rhythm [8]. It is possible that the lack of a metabolic circadian rhythm in the malnourished infants is the result of a lack of sympathetic nervous system stimulation [23]. Reducing or eliminating circadian hormonal stimulation of the sympathetic nervous system may allow more energy to be diverted to rapid growth. This might partly explain the greater energy expenditure in the malnourished infants. The development of a circadian rhythm in infants begins in utero. The suprachiasmatic nuclei, the site of the circadian clock, are present by mid gestation. After birth there are pronounced rhythms in the sleep-wake cycle and hormone secretion by two months of age [24]. Furthermore, fetal heart rate has been found to be synchronized with maternal activity, heart rate, cortisol, maelatonin, and body temperature [25, 26]. Furthermore, the nocturnal trough of body temperature is already present in 6- to 12-week-old full term infants and is a good indicator of the presence of circadian rhythms [27]. All of these studies [25–27] suggest that a metabolic circadian rhythm is present in healthy infants almost from the time of birth.

Another possible explanation for the greater energy intake and expenditure in the malnourished infants during this study may be due to their rapid growth rate. It is estimated that it costs 3.9–6.0 kcal/gram of body weight gain during the first year of life in malnourished infants, representing a 70% energetic efficiency for energy disposition [28]. Considering they have less than half the body fat content and are half the weight of their similarly aged healthy counter parts, this suggests that they are displaying rapid catch-up growth in order to replace their fat-free and fat mass stores back to their genetically predetermined levels. The lack of a metabolic circadian rhythm may also reflect that they are laying down new tissue throughout the day and night. It is possible that their metabolic circadian rhythm might reappear once they reach their normal body weight and composition for their ages.

The EMTAC is the first instrument of its kind to conduct continuous metabolic measurements in infants suffering from primary/secondary malnutrition. Previously, metabolic evaluations in malnourished infants were made utilizing a 20-minute measurement of resting metabolic rate with a Deltatrac metabolic monitor [29]. However, short-term measurements do not encompass the true metabolic needs in these infants due to their unique metabolic profile. In this study where energy expenditure was measured continuously for 22 hours, we found the apparent lack of a metabolic circadian rhythm in the malnourished infants. This might have an effect on the true metabolic needs of these infants. Moreover, the measurement could be repeated and the reappearance of the metabolic circadian rhythm could be possibly used as a sign of recovery.

## 5. Conclusions

This is the first study of its kind in malnourished infants. We found differences in the metabolic profiles between malnourished and healthy infants of similar age. These results will possibly affect determination of future energy requirements and application of nutritional rehabilitation regimes in infants suffering from malnutrition. We also found that they appear to conserve energy, possibly for catch-up growth, though a reduction in physical activity, increased sleep, and the lack of a metabolic circadian rhythm. Moreover, the greater respiratory quotient possibly reflects greater lipid synthesis in order to replace depleted body fat.

## Figures and Tables

**Table 1:** Physical characteristics of malnourished and healthy infants.

	10 Malnourished	10 Healthy [8]
Males/Females	7/3	7/3
Age (months)	7.7 ± 5.6	5.0 ± 0.7
Length (cm)	58.1 ± 5.9*	68.8 ± 2.8
Body weight (kg)	4.1 ± 1.2*	7.3 ± 0.8
Body fat (%)	10.3 ± 7.6*	25.7 ± 2.5
Fat-free mass (kg)	3.6 ± 0.8*	5.4 ± 0.5
BMI (kg/m^2^)	12.0 ± 1.7*	15.4 ± 1.5
Maternal BMI (kg/m^2^)	24.7 ± 4.7	27.1 ± 6.4
Length-for-age z-score	−3.64 ± 1.72*	1.28 ± 1.17
Weight-for-age z-score	−3.78 ± 1.11*	0.26 ± 0.96
Weight-for-length z-score	−1.80 ± 1.01	−1.06 ± 1.00

* = *P* < .05 between malnourished and healthy infants by Independent *t*-test.

**Table 2:** Comparison of 24-hour energy intake, metabolism, and parental interaction between malnourished and healthy infants.

	Malnourished	Healthy [8]
24-hour energy intake (kcal/kg/day)	142.7 ± 14.6*	85.1 ± 25.8
Proportion of energy as carbohydrate (%)	55.1 ± 3.9*	47.2 ± 5.2
Proportion of energy as protein (%)	11.1 ± 1.5	11.0 ± 3.0
Proportion of energy as fat (%)	31.8 ± 9.2	28.3 ± 7.4
24-hour energy expenditure (EE; kcal/kg/day)	101.3 ± 20.0*	78.7 ± 8.4
Sleeping metabolic rate (SMR; kcal/kg/day)	92.6 ± 17.1*	65.0 ± 3.9
24-hour Respiratory Quotient (RQ; VCO_2_/VO_2_)	1.00 ± 0.13*	0.86 ± 0.08
24-hour PA^1^ (Oscillations in weight/minute/kg/body weight)	2.3 ± 0.9*	4.0 ± 1.5
Parental interaction (%)	30.9 ± 8.1	30.6 ± 7.5
Crying time (minutes/day)	133.5 ± 52.5*	88.0 ± 41.8
Sleep (%)	57.2 ± 6.8*	49.7 ± 6.0

* = *P* < .05 between malnourished and healthy infants by Independent *t*-test

^1^PA = Physical activity index.
